# Family burden and caregiver psychological stress in epidermolysis bullosa in China: a cross-sectional survey

**DOI:** 10.3389/fpubh.2026.1851720

**Published:** 2026-05-14

**Authors:** Haoran Kong, Yongchong Chen, Xinglan Guo, Meixuan Liu, Meng Wang, Xiuhong Yang

**Affiliations:** 1 Shandong Medical and Pharmaceutical University, Yantai, China; 2 Beijing Fengtai District Traditional Chinese Medicine Hospital (Beijing Fengtai District Nanyuan Hospital), Beijing, China; 3 Binzhou Healthcare Security Administration, Binzhou, China; 4 Binzhou Medical University Hospital, Binzhou, China

**Keywords:** caregiver burden, cross-sectional survey, epidermolysis bullosa, family caregiving, psychological stress

## Abstract

**Background:**

Epidermolysis Bullosa (EB) is a rare genetic skin disorder requiring long-term intensive care. The disease places considerable economic and caregiving burdens on families, yet factors associated with caregivers’ psychological stress remain inadequately understood.

**Methods:**

A cross-sectional questionnaire survey was conducted among families of patients with EB. Statistical analysis was performed at the household level. In families with multiple affected individuals, the youngest patient was selected as the representative case, as Epidermolysis Bullosa is a rare inherited disorder that predominantly manifests in childhood. This approach was adopted to better reflect the caregiving burden associated with early-onset disease. Data were collected through an online questionnaire. The χ^2^ test was used for comparisons between groups, and ordered logistic regression analysis was performed to identify independent factors associated with high psychological stress among caregivers.

**Results:**

A total of 108 families of patients with EB were included. Children aged ≤6 years accounted for the largest proportion. Families with multiple patients accounted for 7.41%. In 40.74% of the families, the average annual out-of-pocket medical expenses exceeded 50% of the household annual income. In 24.07% of the families, the average daily caregiving time exceeded 4 h. The mean psychological stress score of caregivers was 3.40, and 43.52% reported high psychological stress (≥4 points). Univariate analysis showed that economic burden, caregiving time, and availability of nursing supplies were significantly associated with high psychological stress (*p* < 0.05). However, no statistically significant differences were observed in the ordered logistic regression analysis.

**Conclusion:**

Families of patients with EB experience substantial caregiving and financial burdens. In univariate analyses, caregiving time and nursing supply were associated with caregivers’ psychological stress; however, these variables were not independently significant in the multivariable model. The findings suggest the need for improved insurance coverage, caregiver support, and integrated nursing resources.

## 1 Introduction

Epidermolysis Bullosa (EB) is a group of rare inherited skin disorders characterized by extreme fragility of the skin and mucous membranes. Even minor friction or mechanical trauma can lead to blistering, erosion, and skin detachment (1). In severe cases, multiple organ systems may be involved (2), accompanied by significant complications and increased morbidity and mortality. Based on the level of skin separation and underlying molecular mechanisms, EB is classified into four major types: epidermolysis bullosa simplex, junctional epidermolysis bullosa, dystrophic epidermolysis bullosa, and Kindler syndrome (3, 4). Some patients develop extensive lesions during childhood, leading to a variety of complications, including but not limited to sepsis, anemia, growth retardation, pseudosyndactyly, contractures, and squamous cell carcinoma (5–15). Owing to its rarity and clinical complexity, EB not only imposes a heavy physiological burden on patients but also significantly affects their psychological well-being and social functioning, posing long-term challenges to families and healthcare systems.

Current epidemiological data indicate that the global incidence of EB is approximately 1 in 50,000 live births, while prevalence varies across regions due to differences in diagnostic methods and surveillance systems (16). Patients generally require long-term multidisciplinary supportive care, including wound management, infection control, pain management, and nutritional support. However, current treatment strategies remain largely symptomatic (17, 18), and no curative therapy is currently available (19). The disease also exerts substantial economic and social impacts. High medical expenses and continuous care needs often place considerable financial strain on affected families (18), while family caregivers frequently experience sustained psychological stress (20, 21), time burden (22), and financial pressure (18, 23). Previous studies have mainly focused on the molecular mechanisms, genetic diagnosis, and clinical management of EB, whereas systematic research addressing the disease burden at the family level and the psychological stress experienced by caregivers remains limited (24).

Therefore, this study focuses on families of patients with EB and examines their living conditions, psychological health, and social support. Using a questionnaire-based survey, the study aims to comprehensively evaluate key factors influencing the quality of life of patients and their families. Through systematic data analysis, the research seeks to identify the practical needs of patients in disease management, psychological adjustment, and social integration. Ultimately, this study intends to propose patient-centered support strategies and provide scientific evidence and practical guidance for improving the overall health and social well-being of individuals affected by rare skin diseases.

## 2 Methods

### 2.1 Data collection

This study employed a specially designed questionnaire as the primary tool for collecting basic information from families of patients with Epidermolysis Bullosa (EB), aiming to comprehensively understand the multidimensional characteristics of these families.

### 2.2 Questionnaire

The questionnaire covered several domains, including basic family information, medical and caregiving needs, economic burden, social support, psychological and social needs, daily Care and quality of life in order to ensure the comprehensiveness and representativeness of the data.

The questionnaire was based on literature review and expert consultation, enabling it to accurately reflect the real-life situations of families affected by EB. To improve the response rate and validity of the questionnaire, the research team provided detailed instructions during the distribution process to ensure that participants clearly understood each question.

#### 2.2.1 Ethical governance

Ethical review and approval was not required for this study in accordance with the local legislation and institutional requirements. At the start of the study, the researcher introduced herself and outlined the study’s objectives. Informed consent to participate was obtained from the parents.

#### 2.2.2 Definition

The term “caregiver” referred to the primary family caregiver rather than a professional caregiver. Caregiving time referred to the total daily time spent by the primary family caregiver on EB-related care, including wound dressing, skin care, feeding, bathing, hygiene assistance, and monitoring for complications.

### 2.3 Survey methods

The survey began in March 2025. The distribution and collection of the questionnaire were conducted online to ensure coverage of patient families from different regions and backgrounds. To ensure data accuracy, the research team verified the collected questionnaires after retrieval and excluded incomplete or invalid responses.

### 2.4 Statistical analysis

The data were analyzed using SPSSAU (retrieved from https://www.spssau.com). For analysis purposes, the “Very sufficient” category has been merged with “Sufficient”. This is because “Very sufficient” received a very small number of responses. In all tables and charts, the combined category is reported simply as “Sufficient”. Categorical variables were presented as frequencies and percentages, and comparisons between groups were performed using the χ^2^ test. Ordered logistic regression analysis was conducted to identify factors associated with psychological stress. For multiple-choice questions, the frequency of selection for each option was calculated. All statistical tests were two-sided, and *p* < 0.05 was considered statistically significant.

## 3 Results

### 3.1 Basic characteristics of patients and families

A total of 108 patients with Epidermolysis Bullosa (EB) and their primary caregivers were included in this study. The results showed that the vast majority of families (92.59%) had only one EB patient. Regarding the age distribution of patients, children aged 1–6 years were the largest group (38.89%), followed by those aged 7–12 years (29.63%). Notably, 75.00% of patients had a disease duration of more than 5 years, highlighting the long-term nursing requirements. In terms of caregiving strength, 63.89% of families relied on a single primary caregiver, while only 3.70% had three or more caregivers (Table 1).

**Table 1:** General information of families with EB patients.

Variable	Category	Count	Percentage
Number of EB patients in the household	1	100	92.59
	2	8	7.41
Patient age (years)	<1	4	3.70
	1–6	42	38.89
	7–12	32	29.63
	13–18	16	14.81
	>18	14	12.96
Disease duration (years)	<1	5	4.63
	1–3	13	12.04
	3–5	9	8.33
	>5	81	75.00
Number of primary family caregivers	1	69	63.89
	2	28	25.93
	≥3	4	3.70
	No fixed caregiver	7	6.48
**Total**		108	100.0

### 3.2 Economic and nursing burden

EB families face heavy economic and caregiving pressures. The survey revealed that for 40.74% of families, annual out-of-pocket (OOP) medical and nursing expenses accounted for more than 50% of their total annual household income; for another 37.96% of families, this proportion was between 30 and 50%. Regarding support needs, 75.00% of caregivers identified subsidies for nursing supplies (such as dressings and nutritional supplements) as the most urgently needed form of financial support.

In terms of the nursing burden, 60.19% of caregivers spent an average of 2–4 h per day on patient care. Concerning the supply of nursing products, only 4.63% of families considered the supply to be sufficient, while 38.89 and 24.07% reported that the supply was “insufficient” or “severely insufficient,” respectively. Furthermore, 51.85% of families expressed a need for temporary respite care services but lacked access to them (Table 2).

**Table 2:** Economic and caregiving burden on families.

Variable	Category	Count	Percentage
Patients’ annual out-of-pocket medical and care expenses as a percentage of household income	<10%	4	3.70
	10%–30%	19	17.59
	30%–50%	41	37.96
	>50%	44	40.74
Types of financial support most urgently needed	Family caregiver allowance	18	16.67
	Travel allowance for out-of-town medical treatment	1	0.93
	Subsidies for nursing consumables	81	75.00
	Long-term care insurance	8	7.41
Average daily caregiving time of the primary caregiver (hours)	<2	17	15.74
	2–4	65	60.19
	4–8	15	13.89
	>8	11	10.19
Availability of patients’ daily care supplies	Critically insufficient	26	24.07
	Insufficient	42	38.89
	Generally sufficient	35	32.41
	Sufficient	5	4.63
Level of need for respite care services	Urgently needed	11	10.19
	Needed but currently unavailable	56	51.85
	Not needed at present	41	37.96
**Total**		108	100.0

### 3.3 Psychological stress and support preferences of primary caregivers

The psychological stress levels among caregivers were generally high. On a self-assessment scale of 1 to 5 (with 1 being the lowest), 43.52% of caregivers rated their stress at 4 or 5, and the cumulative proportion of those rating their stress at 3 or higher reached 80.56%. Regarding the choice of psychological support, caregivers showed a strong preference for peer support, with 44.44% opting for “peer support groups,” followed by hospital-based psychological counseling (25.00%) and online support platforms (19.44%) (Table 3).

**Table 3:** Caregivers’ psychological stress.

Variable	Category	Count	Percentage
Primary caregivers’ self-reported psychological stress	1	6	5.56
	2	15	13.89
	3	40	37.04
	4	24	22.22
	5	23	21.30
Preferred types of psychological support	Hospital-based psychological counseling	27	25.00
	Patient peer support groups	48	44.44
	Online support platforms	21	19.44
	Community volunteer intervention	12	11.11
Total		108	100.0

### 3.4 Univariate analysis of factors associated with psychological stress

Chi-square tests were used to analyze the impact of various factors on caregivers’ psychological stress. The results indicated:

Adequacy of nursing supplies: This factor had a significant impact on caregivers’ psychological stress (χ^2^ = 23.221, *p* = 0.026). Among the group with a self-rated stress level of 5, the proportion reporting “severely insufficient” supplies (39.13%) was notably higher than the overall average (24.07%).

Daily care time: This was another significant factor affecting psychological stress (χ^2^ = 23.257, p = 0.026).

Economic burden: Although caregivers in families with a higher proportion of OOP medical expenses generally experienced higher stress, the difference did not reach statistical significance (*p* = 0.083) (Table 4).

**Table 4:** Factors associated with caregivers’ psychological stress.

Variable	Category	Primary caregivers’ self-reported psychological stress					Total	χ^2^	*p*
		1.0	2.0	3.0	4.0	5.0			
Availability of daily care supplies	Critically insufficient	3 (50.00)	2 (13.33)	9 (22.50)	3 (12.50)	9 (39.13)	26 (24.07)	23.221	0.026*
	Insufficient	1 (16.67)	9 (60.00)	12 (30.00)	10 (41.67)	10 (43.48)	42 (38.89)		
	Generally sufficient	2 (33.33)	4 (26.67)	19 (47.50)	8 (33.33)	2 (8.70)	35 (32.41)		
	Sufficient	0 (0.00)	0 (0.00)	0 (0.00)	3 (12.50)	2 (8.70)	5 (4.63)		
Patients’ annual out-of-pocket medical and care expenses as a percentage of household income	<10%	0 (0.00)	0 (0.00)	3 (7.50)	0 (0.00)	1 (4.35)	4 (3.70)	19.242	0.083
	10%–30%	1 (16.67)	7 (46.67)	3 (7.50)	4 (16.67)	4 (17.39)	19 (17.59)		
	30%–50%	1 (16.67)	5 (33.33)	17 (42.50)	12 (50.00)	6 (26.09)	41 (37.96)		
	>50%	4 (66.67)	3 (20.00)	17 (42.50)	8 (33.33)	12 (52.17)	44 (40.74)		
Average daily caregiving time of the primary caregiver	<2	0 (0.00)	4 (26.67)	7 (17.50)	4 (16.67)	2 (8.70)	17 (15.74)	23.257	0.026*
	2–4	3 (50.00)	8 (53.33)	28 (70.00)	14 (58.33)	12 (52.17)	65 (60.19)		
	4–8	0 (0.00)	3 (20.00)	3 (7.50)	5 (20.83)	4 (17.39)	15 (13.89)		
	>8	3 (50.00)	0 (0.00)	2 (5.00)	1 (4.17)	5 (21.74)	11 (10.19)		
Total		6	15	40	24	23	108		

* *p*<0.05; ** *p*<0.01.

### 3.5 Multivariate analysis of factors associated with psychological stress

An ordered logistic regression analysis was conducted with the adequacy of nursing supplies, economic burden, and daily care time as independent variables, and self-rated psychological stress as the dependent variable. The likelihood ratio test showed that the overall regression model was not statistically significant (χ^2^ = 2.263, *p* = 0.520 > 0.05), indicating that within this sample size, the three independent variables did not significantly predict the caregivers’ stress levels (McFadden χ^2^ = 0.007). This finding suggests that caregivers’ psychological stress may be influenced by more complex factors not included in the model, such as the social support system or the clinical severity of the patient’s condition (Table 5).

**Table 5:** Independent predictors of caregivers’ stress in epidermolysis bullosa.

Variable	Var	Regression coefficient	Standard error	*z*	Wald χ^2^	*p*	OR	OR (95% CI)
Dependent variable	1.0	–2.711	0.708	–3.831	14.680	0.000	/	/
	2.0	–1.298	0.619	–2.095	4.391	0.036	/	/
	3.0	0.403	0.605	0.666	0.444	0.505	/	/
	4.0	1.471	0.622	2.367	5.601	0.018	/	/
Independent variable	Availability of daily care supplies	–0.144	0.136	–1.059	1.122	0.289	0.866	0.663–1.130
	Patients’ annual out-of-pocket medical and care expenses as a percentage of household income	0.147	0.148	0.992	0.984	0.321	1.158	0.866–1.548
	Average daily caregiving time of the primary caregiver	0.057	0.165	0.344	0.119	0.731	1.058	0.766–1.463

McFadden *R*^2^ = 0.007;

Cox& Snell *R* ^2^ = 0.021;

Nagelkerke *R* ^2^ = 0.022.

## 4 Discussion

### 4.1 The impact of caregiving demands and resource gaps on caregivers’ mental health

The highly specialized caregiving demands of Epidermolysis Bullosa (EB) are a key driver of caregivers’ psychological stress (25, 26). The present study found that the average daily caregiving time, particularly within the 2–4 h range, was significantly positively associated with caregivers’ self-reported psychological stress scores (*p* < 0.05), indicating a direct transformation of high-intensity physical burden into psychological strain.

Meanwhile, 62.96% of families reported an insufficient supply of nursing materials. Such material shortages not only increase the risk of infection in patients but also objectively intensify the difficulty of caregiving. The limited availability of medical resources, combined with the chronic nature of the disease, places caregivers in a prolonged state of high stress, as they often face the dual challenges of inadequate professional guidance in wound management and insufficient material support (27).

### 4.2 Economic burden as a structural stressor affecting the quality of care

Economic pressure represents a profound structural challenge faced by families of patients with Epidermolysis Bullosa (EB) (28–30) and exerts persistent psychological erosion on caregivers (31, 32). The survey showed that in 40.74% of families, average annual out-of-pocket medical expenditures accounted for more than half of the household’s total annual income. Although some families received financial support from charitable foundations, social organizations, or limited insurance reimbursement, these resources were often insufficient to offset the long-term costs of EB-related care. Such “catastrophic health expenditure” not only severely weakens a family’s capacity to cope with risks but also restricts access to socialized respite services, such as temporary caregiving support.

Financial hardship often leads to social withdrawal and increased caregiver fatigue, creating a reciprocal relationship that further amplifies psychological stress. As a result, the accumulation of psychological stress is not merely an emotional response but rather a socio-psychological outcome arising from the combined effects of high medical expenses, declining quality of life, and insufficient support resources, ultimately hindering the healthy functioning of the family system.

### 4.3 Misalignment of psychological support pathways and the development of a multidimensional intervention system

Psychological stress represents a major challenge for families of patients with Epidermolysis Bullosa (EB) (27, 33, 34). Given the significant imbalance between caregivers’ need for psychological support and the actual utilization of such services, establishing a multidimensional social support system has become imperative (20). The survey found that although 43.50% of caregivers experienced high levels of psychological stress, the utilization rate of professional psychological counseling (25.00%) was substantially lower than that of patient peer support groups (44.44%). This finding highlights the emotional compensatory value of peer-based social support in the management of rare diseases.

In the future, policy-level efforts should focus on optimizing medical insurance reimbursement mechanisms to alleviate financial anxiety. At the service level, the promotion of professional wound care training and the provision of temporary caregiving support should be strengthened. At the societal level, greater support should be provided to patient organizations to establish emotional support networks. Through such integrated support strategies, caregivers’ psychological resilience can be effectively enhanced, thereby improving both the quality of patient care and the overall quality of family life (20).

## 5 Limitations

Although this study provides empirical evidence regarding the survival status of families with Epidermolysis Bullosa (EB), several limitations should be noted. First, due to the low incidence rate of this rare disease, the sample size of 108 cases may result in insufficient statistical power for multivariate regression analysis, which potentially explains the lack of significant findings in the model. Second, the participants were primarily recruited through patient organizations, which may introduce selection bias and fail to fully represent families with limited access to information or those in extremely marginalized situations. Furthermore, the cross-sectional design only reflects psychological stress at a specific point in time, making it impossible to infer causal relationships or dynamically capture the long-term impact of policy adjustments and disease progression on caregivers. Additionally, clinical details such as EB subtype, disease severity, and wound burden were not collected, which limits the ability to assess whether caregiver burden varies by clinical phenotype. Finally, the psychological stress score was based on a self-rated 5-point scale rather than a validated instrument.

## 6 Conclusion

In summary, this study reveals the multiple challenges faced by families of patients with Epidermolysis Bullosa (EB) in terms of living conditions, medical and caregiving needs, economic burden, and social support. It highlights the severity of caregivers’ psychological stress and its impact on the quality of life of patients. These findings not only provide important insights into the real needs of EB patient families but also offer a reference for the development of more effective healthcare policies and support measures in the future.

It is recommended that governments and social organizations increase financial support for families of patients with EB to ensure their access to necessary medical and caregiving resources. In addition, a professional psychological support system should be established to provide counseling and support for caregivers, helping them alleviate psychological stress. Addressing the challenges faced by EB families requires coordinated efforts across three dimensions: material support, economic relief, and psychological reconstruction. Only through such comprehensive approaches can families move beyond merely “maintaining survival” toward achieving a meaningful improvement in their quality of life.

## Data Availability

The raw data supporting the conclusions of this article will be made available by the authors, without undue reservation.
