# Supplementary material for: Family burden and caregiver psychological stress in epidermolysis bullosa in China: a cross-sectional survey
Source: Front Public Health. 2026 May 14;14:1851720. doi: 10.3389/fpubh.2026.1851720 (PMC13216196; doi:10.3389/fpubh.2026.1851720)
Supplement: Supplementary file 1 [file Data_Sheet_1.pdf]

Questionnaire on the Status and Needs of Families with Epidermolysis Bullosa (EB)

I. Basic Information

Number of EB patients in the household: ______

Age of the patient(s): ______ years old

Disease duration:

[ ] < 1 year

[ ] 1–3 years

[ ] 3–5 years

[ ] > 5 years

Number of primary family caregivers:

[ ] 1

[ ] 2

[ ] ⩾ 3

[ ] No fixed caregiver

II. Medical and Nursing Needs

Is the supply of daily nursing products (e.g., sterile dressings, specialized nutritional supplements, non-adherent clothing) sufficient?

[ ] Critically insufficient

[ ] Insufficient

[ ] Generally sufficient

[ ] Sufficient

[ ] Very sufficient

What type of medical support do you need most? (Select all that apply)

[ ] Professional nursing skills training (e.g., wound dressing changes, specialized feeding)

[ ] Guidance on complication prevention (e.g., infection control, nutritional management)

[ ] Telemedicine consultation (online consultation with dermatology/gastroenterology specialists)

[ ] Rehabilitation assistance (physical therapy/functional training)

[ ] Other: __________

III. Economic Burden and Social Support

The patient's average annual out-of-pocket (OOP) medical/care expenses account for approximately ____ of the annual household income:

[ ] < 10%

[ ] 10%–30%

[ ] 30%–50%

[ ] > 50%

Types of assistance already received: (Select all that apply)

[ ] Government medical assistance

[ ] Charitable funds

[ ] Specialized medical insurance reimbursement
[ ] Support from social organizations (e.g., Disabled Persons' Federation)
[ ] No assistance received

What is the most urgent type of financial support you need?

[ ] Subsidies for nursing consumables (dressings, nutritional supplements, etc.)
[ ] Travel allowance for out-of-town medical treatment
[ ] Family caregiver allowance
[ ] Long-term care insurance
[ ] Other: __________

IV. Daily Care and Quality of Life
Average daily time spent on nursing by the primary caregiver:

[ ] < 2 hours
[ ] 2–4 hours
[ ] 4–8 hours
[ ] > 8 hours

Current primary challenges for the family: (Select all that apply)

[ ] Excessive economic pressure
[ ] Restricted social interaction
[ ] Psychological issues among family members (anxiety/depression)
[ ] Stigma and social discrimination
[ ] Home modification needs (e.g., corner guards, constant temperature facilities)
[ ] Educational limitations for the patient (e.g., academic tutoring, special education, education funding)

Level of demand for temporary respite care services:

[ ] Urgently needed
[ ] Needed but currently unavailable
[ ] Not needed at present

V. Psychological and Social Needs
Self-assessment of psychological stress for the primary caregiver (1-5 scale, 1 being the lowest):

[ ] 1 [ ] 2 [ ] 3 [ ] 4 [ ] 5

Preferred psychological support methods:

[ ] Hospital-based psychological counseling

[ ] Peer support groups (patient-to-patient support)
[ ] Online support platforms
[ ] Community volunteer intervention

VI. Long-term Policy Suggestions

Your greatest expectation for support policies for EB families:
